# Intra-articular administration of adrenaline plus an irrigation pump system for visibility during the arthroscopic reconstruction of multiple knee ligaments without a tourniquet

**DOI:** 10.3389/fsurg.2023.1045839

**Published:** 2023-03-17

**Authors:** Xia Zhongyu, Yu Zhen, Guo Bingqing, Kong Xintian, Guo Meifeng, Xu Jianda

**Affiliations:** ^1^ Department of Orthopaedics, Changzhou Traditional Chinese Medical Hospital, Affiliated to Nanjing University of Traditional Chinese Medicine, Changzhou, China; ^2^ Department of Orthopedic, The Third Affiliated Hospital of Soochow University, First People's Hospital of Changzhou, Changzhou, China; ^3^ Department of Acupuncture, Changzhou Traditional Chinese Medical Hospital, Affiliated to Nanjing University of Traditional Chinese Medicine, Changzhou, China

**Keywords:** intra-articular, adrenaline, irrigation pump, visibility, arthroscopy, multiple ligament knee injuries, tourniquet

## Abstract

**Objective:**

Multiple ligament knee injuries (MLKIs) are rare but severe systemic trauma. Single surgery in the acute setting is preferred, although with an extended operation time. To avoid the complications associated with a tourniquet, we herein describe a method for visibility without a tourniquet: intra-articular administration of adrenaline plus an irrigation pump system.

**Study design:**

This is a cohort study with a level of evidence of 3.

**Methods:**

From April 2020 to February 2022, 19 patients with MLKIs were reviewed retrospectively. All patients got intra-articular administration of adrenaline plus an irrigation pump system for visibility without a tourniquet. The following parameters were assessed: visibility, range of motion, knee stability, visual analog scale (VAS) score, range of motion (ROM), Lysholm score, Tegner activity level, and International Knee Documentation Committee Subjective Knee Form (IKDC).

**Results:**

All patients were followed up for at least 6 months. At the latest follow-up, the mean VAS score, ROM, Lysholm score, and IKDC were 1.79 ± 0.86, 121.21 ± 10.96, 88.16 ± 5.21, and 88.53 ± 5.06, respectively. The average Tegner activity level decreased significantly from preinjury to postoperation (5.16 ± 0.83 vs. 3.11 ± 0.88, *P* < 0.001). Of the 19 patients, 17 (89.47%) had good knee function, while only two patients (10.53%) had asymptomatic knees with positive Lachman tests. A total of 17 patients (89.47%) had good or excellent visualization during arthroscopy. Of the 19 patients, three (15.79%) required an increased fluid pressure to achieve a clear operative view. Two patients converted to tourniquet inflation due to persistent intra-articular bleeding after using shavers.

**Conclusions:**

The intra-articular administration of adrenaline plus an irrigation pump system is recommended as an alternative to a tourniquet to achieve a good visual field. Further evidence-based work with a larger sample is needed.

## Introduction

Multiple ligament knee injuries (MLKIs) are rare but severe systemic trauma and include knee dislocations and disruption of multiple structures, such as ligaments, capsules, menisci, bone, blood vessels, and nerves (1). Although there has been some controversy regarding single-stage or multistage arthroscopic surgery, recent systematic reviews have confirmed that acute single-stage intervention (<3 weeks) could improve the functional prognosis (2–4). These concomitant injuries are potentially devastating and extend operation time.

Adequate visualization is key to safe and effective arthroscopic procedures. Although various inflation pressures are reported, a tourniquet is regularly used to improve operative field visibility in knee arthroscopy. A meta-analysis concluded that using a tourniquet could improve visualization during arthroscopic knee surgery (5). However, tourniquet-related complications are not uncommon, especially in patients with repeated use during one operation. If the operation time is longer than 60 min, using a tourniquet could increase complications, including electromyographic changes, decreased muscle endurance, and functional muscle weakness (6). Limiting use to key parts of an operation may be of benefit.

The most common tourniquet-related complication is nerve injury due to mechanical compression and neural ischemia (7). Other following postoperative complications include vascular injury, pain, decreased muscle endurance, and functional weakness. Olivecrona et al. demonstrated that extended tourniquet time significantly increased the risk of superficial infection (8). It is better to reduce the time of a tourniquet or not use it.

To avoid the complications associated with a tourniquet, we herein describe a method for visibility without a tourniquet: intra-articular administration of adrenaline plus an irrigation pump system.

## Patients and methods

From April 2020 to February 2022, 19 patients with MLKIs were reviewed retrospectively and followed up (Table 1). There were 11 men (57.89%) and eight women (42.11%) with an average age of 46.42 ± 11.22 years (range, 28–60 years). The inclusion criteria were patients with unilateral MLKIs, single-stage intervention, complete clinical data, and no other prior diseases affecting knee function. Some patients who had bilateral or other prior diseases affecting knee function, had received staged interventions, or had incomplete clinical data were excluded.

**Table 1:** Characteristics of patients.

Patients	Age/gender	Mechanism	Injured ligament structures	Meniscus	Fracture	ROM	Follow-up (months)	VAS	Lysholm score	IKDC
1	28/M	Vehicle	APM	Y	N	130	11	1	95	84
2	55/F	Vehicle	APM	Y	N	125	10	3	85	90
3	55/F	Sport	APM	Y	N	140	8	1	90	91
4	36/M	Vehicle	APL	Y	N	125	6	2	80	94
5	38/M	Vehicle	APM	N	N	120	6	1	93	80
6	36/F	Vehicle	APM	Y	Y	110	21	2	94	82
7	30/M	Vehicle	APM	Y	N	103	20	1	85	84
8	60/M	Vehicle	APL	Y	N	126	19	3	90	93
9	40/F	Vehicle	APM	Y	N	122	20	1	83	94
10	55/F	Vehicle	APM	Y	N	124	15	3	92	90
11	35/M	Sport	APM	N	N	116	15	2	88	87
12	54/M	Vehicle	APM	Y	N	125	16	2	93	96
13	56/F	Vehicle	APM	Y	Y	99	12	1	87	85
14	59/M	Vehicle	APM	Y	Y	104	14	3	90	82
15	49/M	Vehicle	APM	Y	N	116	13	1	92	88
16	31/F	Fall	APL	Y	Y	128	13	3	75	84
17	57/M	Vehicle	APM	Y	N	123	27	1	83	90
18	56/F	Vehicle	APM	Y	N	132	28	1	90	91
19	52/M	Vehicle	APM	Y	N	135	27	2	90	97

VAS, visual analog scale; IKDC, International Knee Documentation Committee Subjective Knee Form; ACL, anterior cruciate ligament; PCL, posterior cruciate ligament; MCL, medial collateral ligament; LCL, medial or lateral collateral ligament; Y, Yes; N, No; ROM, range of motion.

APM, ACL + PCL + MCL; APL, ACL + PCL + LCL.

## Surgical technique

All operations were performed under general anesthesia by the same senior experienced orthopedic surgeon specialized in arthroscopy. The patients were routinely placed in the supine position with the lateral thigh supported by a solid baffle. A foot pad was placed under the foot to keep a 90° flexion position, which could be changed if needed. The tourniquet was routinely placed on the thigh prior to skin preparation in case of poor hemostatic effect during the operation. Bony landmarks and portal placements were marked preoperatively. A systematic inspection was performed once the cavity of the knee was entered.

The sequence of graft fixation depended on the involved ligament reconstructions. The torn ligaments of the anterior cruciate ligament (ACL)/posterior cruciate ligament (PCL) were preferably reconstructed with the unilateral or bilateral peroneal longus tendon. The medial or lateral collateral ligament (MCL/LCL) was preferably repaired using the open surgical technique. A suture anchor combined with a lateral anchor suture (Healix Advance Knotless, DePuySynthes) was used if the avulsion fracture of the lateral collateral ligament occurred. In patients with LCL reconstruction, the fixation of LCL reconstruction grafts was performed after the PCL but before the ACL fixation. The treatment choice (suture or partial meniscectomy) for meniscal injuries was based on the individual injury. The fourth-degree injury of the articular cartilage was treated with the microfracture technique. Ten milliliters of the analgesic regime (5 mL of ropivacaine + 5 ml of triamcinolone acetonide) was injected into the joint. Any intraoperative visualization difficulties, conversion to tourniquet inflation, and complications were recorded.

## No-tourniquet regime

Before the procedure, 1 mL of adrenaline (1:1,000) was diluted in 50 mL of normal saline. After sterilization, 10 mL of adrenaline solution was infiltrated into the portal sites. The other adrenaline solution was infiltrated into the knee joint immediately. Repeated flexion and extension of the knee were performed to achieve better drug distribution and hemostatic effect.

An irrigation pump system (CONMED Corporation, Utica, NY, United States) with normal saline fluid was used in each patient. The pressure was regularly set to 35 mmHg and increased in increments of 5 mmHg if needed.

## Physiotherapy regime

Isometric muscle training was performed after the operation. A hinged knee brace was used to protect the stability of the injured knee and overcome varus and valgus stress. Patients participated in rehabilitation training twice a day. Passive flexion and extension activities were encouraged according to individual tolerance from the second day after the operation. The rehabilitation goal within 2 weeks was to achieve a ROM of 90°. Weight-bearing on the affected limb was prohibited until 3–4 weeks later, whether the meniscus was sutured or not. The patients gradually transitioned from partial weight-bearing to full weight-bearing.

## Evaluation parameters

The visual analog scale (VAS) score was used to assess pain intensity on a scale from 0 (no pain) to 10 (worst pain) using a 10-cm horizontal line (9).

The Lysholm score (10), Tegner activity level (11), and International Knee Documentation Committee Subjective Knee Form (IKDC) (12) were used to evaluate the functional results.

The range of motion was measured using goniometry.

Knee stability was assessed using the Lachman test, which was employed to evaluate the postoperative laxity of the injured knee.

The surgeon was asked about the visibility by combined score of visibility and ease of procedure (poor, fair, good, and excellent) (13).

## Statistics methods

All statistical analyses were performed using SPSS V.24.0 (SPSS Inc., Chicago, IL, United States). The normal distribution was assessed by the Kolmogorov–Smirnov test. Means and standard deviations were used for normal continuous variables, and medians with interquartile ranges were used for non-parametric continuous variables. A paired sample *T*-test was used to compare Tegner activity levels from preinjury to postoperation. *P* < .05 was considered statistically significant.

## Results

All patients received single-stage arthroscopic surgery and were followed up for at least 6 months. Of them, four patients had accompanying fractures of the tibial plateau. The microfracture technique was used in three patients with fourth-degree injuries of articular cartilage. A total of 16 patients had multiple ligament injuries of ACL + PCL + MCL, while the others had injuries of ACL + PCL + LCL. At the latest follow-up, the mean VAS score, ROM, Lysholm score, and IKDC were 1.79 ± 0.86, 121.21 ± 10.96, 88.16 ± 5.21, and 88.53 ± 5.06, respectively. The average Tegner activity level decreased significantly from preinjury to postoperation (5.16 ± 0.83 vs. 3.11 ± 0.88, *P* < 0.001). On clinical examination of knee stability, 17 of the 19 (89.47%) patients had good knee function, while only two patients (10.53%) had asymptomatic knees with positive Lachman tests.

Of the 19 patients, 17 (89.47%) had good or excellent visualization during arthroscopy. Of the 19 patients, three (15.79%) required an increased fluid pressure to achieve a clear operative view. Two patients converted to tourniquet inflation due to persistent intra-articular bleeding after using shavers. It made the no-tourniquet procedure impossible and it was, therefore, abandoned.

There were no systemic complications, such as heart rate or mean arterial pressure variabilities, pulmonary edema, and cardiopulmonary arrests, or local complications, such as compartment syndrome and neurovascular compromise. The wounds in two patients had fat liquefaction, and healed after dressing changes. One patient complained of mild pain associated with climatic changes around her knee joint after the operation.

## Discussion

In this study, we confirmed that intra-articular administration of adrenaline plus an irrigation pump system was safe to establish a clear arthroscopic view during the operation. Also, no serious complication was found during the perioperative period.

A tourniquet is regularly used to improve operation field visibility in knee arthroscopy. Hoogeslag et al. conducted a randomized double-blinded trial and indicated that using a tourniquet supplied better visibility for routine knee arthroscopy but did not reduce the operation time (14). However, tourniquet-related complications are not uncommon. When used inappropriately, the direct mechanical pressure from tourniquets can lead to systemic upset, even permanent morbidity. The duration of knee arthroscopy has been regarded as the only risk factor for complications (15). The current view is that increased tourniquet time and increased tourniquet pressure are the main risk factors for tourniquet-related complications (16). There is still a debate about the “safe” tourniquet time due to little evidence in the literature. Most researchers accept that, based on animal and human studies, the maximum tourniquet time is 2 h. A deflation interval with an hourly release of 10 min is needed if the tourniquet time is longer than two hours (17). Tourniquet use duration of less than 30 min does not induce muscle damage that hinders the opportunity for light work. It did not increase postoperative pain or cause a recurrence of pain after arthroscopic meniscectomy (18). Therefore, tourniquet is always limited used in the key procedures of operation.

Single-stage arthroscopic surgery for patients with MLKIs usually needs a longer operation time with repeated use of a tourniquet. Considering the tourniquet-related complications, many surgeons have been trying to explore new alternative methods to tourniquets. Johnson et al. performed a prospective study and concluded that knee arthroscopy did not routinely require a tourniquet to achieve a clear operative view (19). In the present study, we described a method for visibility without a tourniquet: intra-articular administration of adrenaline plus an irrigation pump system. Surgeons felt that, on average, the no-tourniquet regime supplied good intraoperative visibility, avoiding tourniquet inflation.

Intra-articular injection of epinephrine can supply a bloodless field due to its vasoconstrictive action. It reduces local perfusion and creates a “tourniquet-like” effect on the knee joint. Portal site bleeding could be reduced significantly by local adrenaline solution infiltration before incision. Epinephrine was confirmed to improve surgeon-rated visualization in shoulder arthroscopy, but it did not decrease operation time (20). Jansen and White infiltrated 1 mL of adrenaline (1:1,000) into the hip joint and left it inside for 1 min to diminish intra-articular bleeding. The visual field clarity during arthroscopy and additional portal placement was noted to be better (13). Local adrenaline infiltration provided good hemostasis in the joint capsule and synovium in wrist arthroscopy (21). Additionally, a pressure pump further helps hemostasis and obviates the routine use of a tourniquet. Karaoglu et al. pointed out that adding epinephrine to the portal incisions alone was sufficient for a good operative view. Also, the heart rate and mean arterial pressure did not change significantly (22). The previous literature had described the same conclusions that subcutaneous and intra-articular injection of epinephrine established a sufficiently bloodless field during routine arthroscopic anterior cruciate ligament reconstruction surgery (23). This study draws the same conclusion.

Epinephrine is toxic to articular cartilage and synovial tissue in a dose- and time-dependent manner. Rao et al. added 1:1,000 epinephrine to human chondrocytes and synovial cells for 90 min and concluded that it induced chondrocyte and synovial cell apoptosis in a time-dependent manner (24). Human chondrocytes were treated with different doses of epinephrine solutions. The results showed that low-dose epinephrine was less toxic to human articular chondrocytes *in vitro* (25). The potential risk of chondrolysis was reported due to the cytotoxic effects of epinephrine on chondrocytes (26, 27). In the present study, we observed that the instar-capsular concentration of adrenaline did not reach the potentially toxic concentration. The exposure time to articular cartilage is short. Of course, a second arthroscopy is needed to confirm this conclusion in the future.

An additional irrigation pump system was used to keep intra-articular pressure at 35 mmHg to reduce discrete bleeding points and improve the operative view. A pressure pump can overcome venous and/or capillary pressure (28). The fluid pressure necessary for bleeding control in small veins is 28 mmHg. Therefore, the pressure was regularly set to 35 mmHg. Excessive intra-articular pressure may result in synovial pouch tear and fluid extravasation into the calf. The position of the knee should be changed gently to avoid free backflow into the inflow system for excessive intra-articular pressure (29). No related complication was found during the perioperative operation.

## Limitations

The present study has several limitations. First, it is just a retrospective study of 19 patients; a well-designed prospective randomized controlled trial with larger sample size is needed. Second, to achieve a good surgical view with minimum toxicity, the safe threshold of pump pressure and proper adrenaline dose need to be explored in further studies. A long-term follow-up on the effects of epinephrine when in direct contact with the articular cartilage is also needed.

## Conclusion

Overall, the current study shows that good visual field clarity can be achieved without a tourniquet for patients with MLKIs. The intra-articular administration of adrenaline plus an irrigation pump system is recommended as an alternative. Further evidence-based work with a larger sample is needed.

## Data Availability

Publicly available datasets were analyzed in this study. All data generated or analyzed during this study are included in this published article and are available from the corresponding author upon reasonable request.
